# Mapping Horizontal Spread of Activity in Monkey Motor Cortex Using Single Pulse Microstimulation

**DOI:** 10.3389/fncir.2016.00104

**Published:** 2016-12-16

**Authors:** Yaoyao Hao, Alexa Riehle, Thomas G. Brochier

**Affiliations:** ^1^Institut de Neurosciences de la Timone, CNRS – Aix-Marseille Université, UMR7289Marseille, France; ^2^RIKEN Brain Science InstituteSaitama, Japan; ^3^Institute of Neuroscience and Medicine, Forschungszentrum JülichJülich, Germany

**Keywords:** multi-electrode array, single unit activity, multi unit activity, cortical mapping, cortical connectivity

## Abstract

Anatomical studies have demonstrated that distant cortical points are interconnected through long range axon collaterals of pyramidal cells. However, the functional properties of these intrinsic synaptic connections, especially their relationship with the cortical representations of body movements, have not been systematically investigated. To address this issue, we used multielectrode arrays chronically implanted in the motor cortex of two rhesus monkeys to analyze the effects of single-pulse intracortical microstimulation (sICMS) applied at one electrode on the neuronal activities recorded at all other electrodes. The temporal and spatial distribution of the evoked responses of single and multiunit activities was quantified to determine the properties of horizontal propagation. The typical responses were characterized by a brief excitatory peak followed by inhibition of longer duration. Significant excitatory responses to sICMS could be evoked up to 4 mm away from the stimulation site, but the strength of the response decreased exponentially and its latency increased linearly with the distance. We then quantified the direction and strength of the propagation in relation to the somatotopic organization of the motor cortex. We observed that following sICMS the propagation of neural activity is mainly directed rostro-caudally near the central sulcus but follows medio-lateral direction at the most anterior electrodes. The fact that these interactions are not entirely symmetrical may characterize a critical functional property of the motor cortex for the control of upper limb movements. Overall, these results support the assumption that the motor cortex is not functionally homogeneous but forms a complex network of interacting subregions.

## Introduction

Exploring the functional organization of motor cortex from the perspective of intracortical connectivity is fundamental to understand how cortical neurons control movement. Intracortical microstimulation (ICMS) mapping studies have shown that distinct areas of the motor cortex are, respectively, involved in the descending control of the face, arm, and leg following a basic somatotopic organization, classically described by Woolsey et al. (1952). Complementary studies revealed that the concept of somatotopic organization is less appropriate to describe the intrinsic organization of each body area. For instance, within the upper limb area, there is no strict border between the cortical sites evoking ICMS responses in the elbow, wrist, and digits suggesting that these upper limb body parts are controlled by broadly overlapping cortical territories (Murphy et al., 1978; Schieber, 2001; Graziano and Aflalo, 2007; Boudrias et al., 2010). In line with these observations, anatomical studies revealed a dense network of horizontal connections linking distant cortical points of the motor cortex (Huntley and Jones, 1991; Keller, 1993; Weiss and Keller, 1994). In rodent, cat, and macaque monkey, these intrinsic connections preferentially link representation zones within the face, leg, or arm areas but are missing between these distinct body areas. Retrogradely labeled cell bodies following horseradish peroxidase (HRP) injections in the digit representation are distributed within the digit representation as well as in the wrist, elbow, and shoulder representations but they are nearly absent in the face and lower limb areas (Huntley and Jones, 1991; Keller, 1993). In addition, it has been suggested that in the macaque monkey, the connection terminals show a patchy distribution preferentially aligned along the anterio-posterior axis linking digit and arm related areas, respectively (Huntley and Jones, 1991). In a more recent study, Capaday et al. (2009) used the anterograde tracer biocytin and quantified precisely the density of synaptic buttons along horizontal collaterals in the cat motor cortex. In contrast to earlier studies, they observed a dense labeling within a 1–2 mm radius around the injection site and, beyond this core, a monotonic decrease in button density with distance. The horizontal connections are broadly distributed in space, toward intermingled proximal and distal upper limb representations (Capaday et al., 2009).

Since these anatomical studies only highlight the pattern of monosynaptic connections and may underestimate the full complexity of *trans*-synaptic network interactions, some functional approaches have been used to characterize in more details the intrinsic organization of the motor cortex. Functional studies classically combine ICMS techniques with extracellular recordings of neuronal responses to the stimulus to explore the properties of activity propagation in the motor cortex. Single pulses of ICMS (sICMS) evoke robust responses in neurons recorded up to 2 mm away from the stimulation site (Asanuma and Rosen, 1973; Aroniadou and Keller, 1993; Matsumura et al., 1996; Baker et al., 1998). The first component of these stimulus-evoked responses is mainly excitatory, commonly followed by a period of activity suppression, suggesting that the neuronal activity propagates through a cortical network of excitatory and inhibitory interneurons (Hirsch and Gilbert, 1991; Schubert et al., 2001; Isaacson and Scanziani, 2011). Capaday et al. (2011) analyzed the spatiotemporal property of activity spread by implanting multielectrode arrays in the cat motor cortex. Using a global measure of multinunit activity, they observed that local activation of the cortex by bicuculline injection (a GABA_A_ antagonist) generates a spread of activity symmetrically distributed around the site of stimulation and invading a large cortical territory (∼7 mm^2^) at an average velocity of 0.14 m/s. Other studies analyzed the spread of neuronal activity triggered by external visual/auditory stimuli (Stevenson et al., 2009; Field et al., 2010; Muller et al., 2014) or the spiking activity of single neurons (Zanos et al., 2008, 2011; Nauhaus et al., 2009, 2012). In particular, based on spike-triggered averaging of the LFP recordings, Nauhaus et al. (2009) demonstrated outward waves of propagation traveling for several millimeters along the cortical surface.

In the present study, we combine ICMS and extracellular recordings using chronically implanted multielectrode arrays to explore the spatiotemporal properties of activity spread in the motor cortex of the awake macaque monkey. We aim at revealing some key principles of motor cortex organization by analyzing the extent and orientation of activity spread from distinct locations on the cortical surface. An artifact reduction protocol enabled recordings of single unit (SUA) and multiunit (MUA) activity within less than 1 ms after stimulation. The spatiotemporal property of horizontal propagation was systematically mapped in the intermediate layers of the motor cortex over a surface of several square millimeters. We found that the amplitude of the stimulation effect is primarily modulated by the distance to the stimulating electrode and approximated by an exponential function. Excitation effects extend to longer distances than inhibition effects. The spatial distribution of the spread varies from electrode to electrode and demonstrates long distance tuning for preferred orientations. Altogether, these observations provide novel information about the complex topological organization of intrinsic motor cortical connections in non-human primates, and refine the understanding of motor cortex organization in relation to upper limb movements.

## Materials and Methods

Two adult monkeys (Macaca mulatta, monkey S and N, female and male, respectively) were used in this study. Monkey N has been trained and recorded in an instructed-delay reach-to-grasp task (see for the task description Riehle et al., 2013) before the experiment, while monkey S was naïve. The experiment was performed 5 and 6 months after array implantation and the data presented in this study were collected over a 10- and 6-day period for Monkey N and S, respectively. All animal procedures were approved by the local ethical committee (authorization A1/10/12) and conformed to the European and French government regulations.

### Microelectrode Array Implantation

**FIGURE 1:**
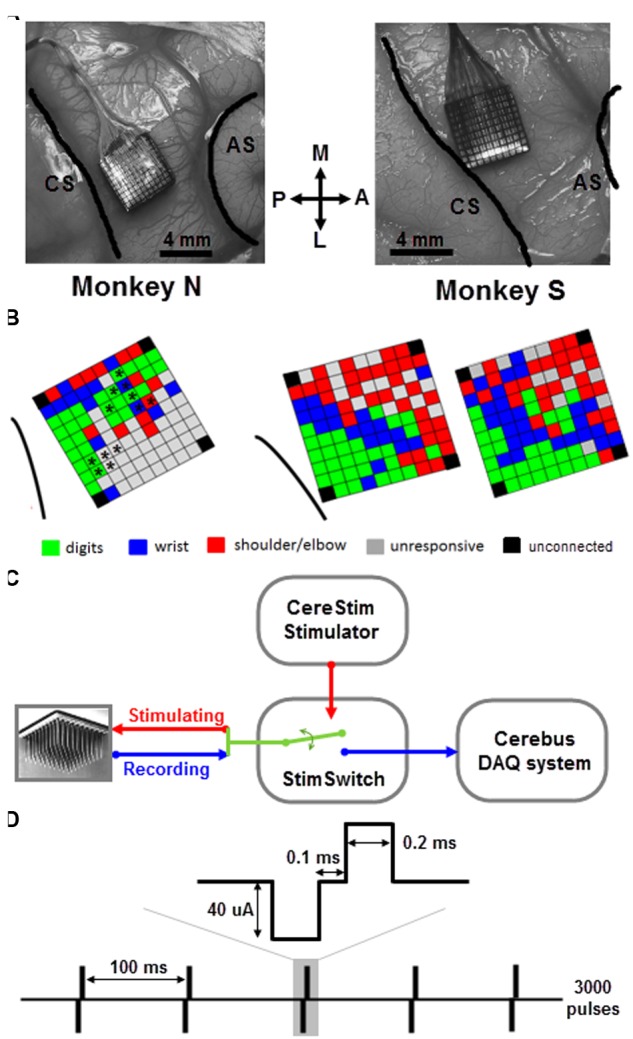
**Array implantation and stimulation/recording setup. (A)** Utah arrays were implanted into the motor cortex of the right hemisphere of two monkeys (N and S) between central sulcus (CS) and arcuate sulcus (AS). M, medial; L, lateral; A, anterior; P, posterior. **(B)** Map of ICMS effects evoked at each electrode of the array in monkey N (left) and S (right) using the classical ICMS protocol (Asanuma and Rosen, 1973; see text for more details). For monkey N, the asterisks indicate the subset of electrodes used for sICMS. For monkey S, the two maps were tested in two independent sessions 3-month apart. Note, that in both monkeys the four corner electrodes were not connected. In case of multi-joint response at a given electrode, the most distal response is shown on the map. **(C)** CereStim stimulator and Cerebus recording system were connected to the electrode array through a switch (StimSwitch, Blackrock microsystems), enabling to switch each electrode between stimulating or recording mode. **(D)** The single pulse stimulation pattern consisted of 3000 pulses (0.2 ms biphasic, cathode first, 40 μA) delivered at 10 Hz (i.e., interpulse interval = 100 ms).

Monkeys were implanted with a 100-electrode Utah array (Blackrock Microsystems, Salt Lake City, UT, USA) in the motor cortex of the right hemisphere (**Figure 1A**). The 4 mm × 4 mm silicon based array consisted of 10 × 10 Iridium-Oxide electrodes, of which 96 were available for electrical recording and stimulation. The length of each electrode was 1.5 mm, with a 400 μm inter-electrode spacing. With this electrode length, we assume that the array enabled recording and stimulation between the deep cortical layer III and the most superficial part of layer V. The distance between any pair of electrodes can be easily determined from the fixed geometric structure of the array. The surgery for array implantation was described before (Riehle et al., 2013) and is briefly summarized below. The surgery was performed under deep general anesthesia using full aseptic procedures. A 30 mm × 20 mm craniotomy was performed over the motor cortex and the dura was incised and reflected. The array was inserted into the motor cortex between the central and arcuate sulci (**Figure 1A**) using a pneumatic inserter (Blackrock Microsystems). It was then covered by a non-absorbable artificial dura (Preclude, Gore-tex). Ground and reference wires were inserted into the subdural space. The dura was then sutured back and covered with a piece of artificial absorbable dura (Seamdura, Codman).

The bone flap was put back at its original position and secured to the skull by a titanium strip and bone screws (Codman). The array connector was fixed to the skull on the hemisphere opposite to the implant. The skin was sutured back over the bone flap and around the connector. The monkey received a full course of antibiotics and analgesics after the surgery and recovered for 1 week before the first recordings.

### ICMS Mapping

We used a standard ICMS approach to map the somatotopic organization of the cortical area covered by the multi-electrode array (Asanuma and Rosen, 1973). We identified movements of the upper limb evoked by trains of 15 biphasic pulses delivered at 300 Hz, (cathodal first, 200 μs width for each phase) applied successively at each electrode using a 96-channel programmable stimulator (Cerestim96, Blackrock Microsystems). Two experimenters were involved in this part of the experiment. The first experimenter operated the stimulation software and logged the motor response evoked at each stimulation site. The second experimenter held the monkey’s arm and determined the motor response by visual inspection of the twitching movement evoked at the digit, wrist, and arm joints and by muscle palpation. No EMG was recorded. Trains of ICMS were triggered manually at about 0.5 Hz and the current intensity was adjusted such to evoke movements at the threshold level in a consistent way. We started at a low intensity of 20 μA and gradually increased up to 100 μA with a step of 10 μA. If there was no obvious movement evoked at the highest intensity, the electrode was defined as unresponsive. We distinguished between movements evoked at the digits, wrist, and shoulder/elbow by visual inspection of the arm during ICMS and to produce a basic map of ICMS effects for each array (**Figure 1B**). For a few electrodes (7 out of 96 for monkey S), the evoked response at threshold intensity spanned multiple joints (usually finger/wrist or wrist/elbow). The multi-joint responses are likely to reflect the activation of wrist or extrinsic hand muscles crossing the elbow and acting both on the proximal and distal joints. For these responses, we therefore indicated the movement evoked at the most distal joint in the ICMS maps of **Figure 1**.

### Electrical Stimulation and Recording

The cortical spread of stimulation-evoked activity was analyzed by applying sequences of sICMS at each electrode one by one, while the evoked responses were recorded at all other electrodes (Kraskov et al., 2011). Each pulse was ground referenced, biphasic, rectangular and charge balanced (200 μs width for each phase) with the cathodal pulse preceding the anodal. During a typical sICMS sequence, single pulses were repeated at a rate of 10 Hz for a total number up to 3000 pulses (**Figure 1C**). In both monkeys, we commonly used a stimulation intensity of 40 μA, although a range of intensities between 10 and 80 μA (by increasing steps of 10 μA) was also tested. The sICMS protocol was applied to each electrode in monkey S but only to at a subset of electrodes in monkey N (**Figure 1B**). During stimulation of each electrode, neural data from all 96 channels were recorded using the Cerebus system. Note, that in both monkeys the four corner electrodes were not connected. The signals were buffered (unity gain headstage, Patient Cable, Blackrock Microsystems), amplified (Blackrock Front End Amplifier, gain x5000) and filtered (bandpass, 0.3–7500 Hz), before digitizing at a sample rate of 30 kHz. No further digital processing was applied during recording and the band-passed raw signal was stored for oﬄine analysis. Trigger signals from the stimulator were stored along with the neuronal signals. The monkey was awake throughout the stimulation, quietly sitting in a custom-made primate chair with no head and arm constraint. The typical sICMS sequence (3000 pulses, 10 Hz) lasted 5 min such that only a subset of electrodes (5–20) could be tested on a single day. Several consecutive days of recording were necessary to run the sICMS protocol at all electrodes.

### Artifact Reduction and Signal Extraction

**FIGURE 2:**
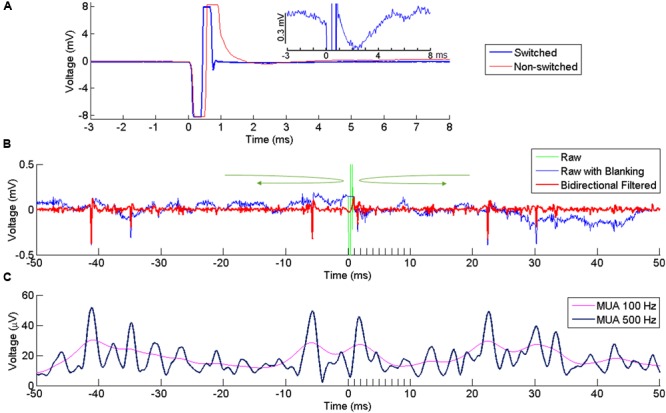
**Artifact reduction and extraction of neural signal. (A)** Artifact at a recording channel 400 μm away from the stimulating channel with (blue) our without (red) switching between stimulating and recording modes. Time 0 indicates the onset of stimulation. The stimulation lasts for ∼ 0.5 ms, resulting in the first negative phase of the artifact. In the non-switched mode, the artifact lasts for more than 0.5 ms after the offset of stimulation due to saturation of the amplifier. The long tail of the artifact is suppressed in the swicthed-mode, allowing fast recovery of neural activity after the stimulation offset. The inset plot shows the response in the switched mode at higher amplitude resolution. **(B)** Superimposed broad-band signal with the stimulation artifact (green), broad-band signal after artifact replacement (blue) and its high-pass filtered version (red) following bidirectional filtering separately applied to the pre-stimulus and post-stimulus signal, in the direction indicated by the green arrows. A clear spike is visible right after stimulus offset. **(C)** MUA computed from the signal in **(B)**, using two different cutoff frequencies (100 and 500 Hz).

Following ICMS, the signal recorded at neighboring electrodes is contaminated by a large stimulus artifact whose amplitude increases with the stimulation intensity. In order to reduce contamination of the recorded signals by the artifact, we used a PC-controlled 128-channel electrode switch (StimSwitch, Blackrock Microsystems, Salt Lake City, UT, USA) that switches each individual channel between stimulating and recording mode. The CereStim stimulator generates a TTL signal that switches pre-selected channels to the stimulation mode 60 μs before passing the stimulation current to the electrodes (**Figure 1D**). The TTL signal stops once the stimulation sequence is completed and turns the channels back into the recording mode. As shown in **Figure 2A**, the signal recorded next to the stimulating electrode in the non-switched mode (red) showed a long artifact tail exponentially decreasing after the first ms of amplifier saturation. This artifact prevented recording of neural activity within 2–3 ms after stimulation onset. The signal recorded in the ‘switched’ mode (blue) also showed an early artifact during stimulation due to the channel switching to the high-impedance stimulator end. However, after saturation, the signal returned rapidly to baseline due to the fast discharge of the stimulating electrode when switched back to recording mode. As a result, the duration of the artifact never exceeded 1 ms on all recording channels and clear neural activity could be recorded as early as 1 ms after stimulation onset.

However, even after hardware switching, the stimulus-evoked artifact recorded in the broadband signal had much larger amplitude than a standard electrophysiological signal (Wagenaar and Potter, 2002). Any filtering applied to this broadband signal would lead to a contamination of the neural signal around the time of the artifact. To avoid this contamination, we excluded the artifact window from the filtering process by filtering the signal separately before and after the artifact using a bidirectional band-pass filter (4th order Butterworth, 250–5000 Hz for SUA, 300–6000 Hz for MUA). The bidirectional filtering was applied forward-first and backward-first before and after the artifact, respectively (green arrows in **Figure 2B**). As the blanking window itself was not included in the filtering, the signal around the window was uncontaminated by the artifact and could be used to extract SUA and MUA from the recording channels.

To extract SUAs, the time window containing the stimulation artifact (i.e., from 0 to 1ms after stimulus onset) in the band-pass filtered signal was replaced by a constant voltage with a value corresponding to the last voltage measured before stimulus onset (**Figure 2B**). This artifact-free signal (time resolution 30 kHz) was then imported in a spike-sorting software (Oﬄine Sorter, version 3.3.3, Plexon Inc., Dallas, TX, USA) to extract the timing of single unit responses. To include only large and clear SUA in our analysis, we set a threshold at -8 ^∗^ standard deviation (SD) and all waveforms crossing this threshold were sorted and clustered in the PCA space. In the present study, we only analyzed SUAs that were stable enough to be isolated over several recording sessions and days in which all other electrodes were used in turn as stimulating electrode.

MUAs were estimated using a conventional root mean square (RMS) method (Stark and Abeles, 2007). MUA was performed by clipping extreme values (larger or smaller than the mean ± 2 ^∗^ SDs) of the filtered signal and computing the sample-by-sample RMS. A low-pass filter at 100 Hz is commonly used for the RMS (Stark and Abeles, 2007; Capaday et al., 2011) but we opted for a 500 Hz low-pass filtering to enhance the temporal resolution of the MUA signal. With these settings, both high and low amplitude single units contributed to the modulations observed in the MUAs (**Figure 2C**).

### Spatiotemporal Analysis of SUA and MUA Responses

**FIGURE 3:**
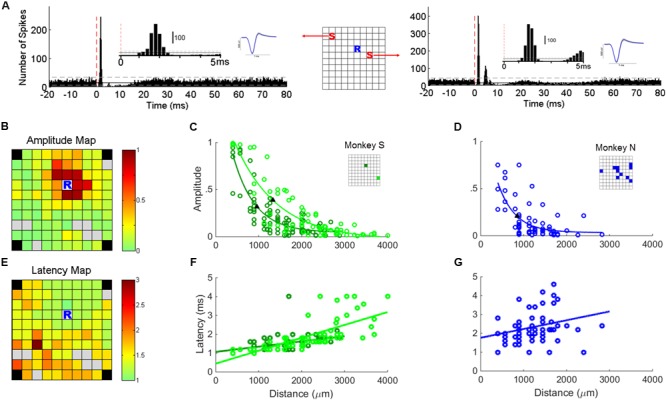
**Single unit responses to sICMS. (A)** The PSTHs of one typical unit recorded at location ‘R’ in response to stimulations at location ‘S’ in the schematic representation of the array (center). The red dashed line indicates the stimulation onset (time 0). The insets show the first 5 ms of the PSTH and the average waveform of the SUAs. **(B)** Map of the array showing the amplitude of the SUA response recorded at ‘R’ while sICMS is applied at all other electrodes. The amplitude of the response is color-coded at the location of the stimulating electrode (same SUA as in **A**). Gray squares indicate electrodes whose evoked response was not significant. Note, that in both monkeys the four corner electrodes were not connected. **(C)** Relationship between response amplitude and distance for the stable SUA of monkey S. The locations of the two recorded units are indicated in the inset grid. **(D)** Relationship between response amplitude and distance for all possible combinations of stimulating electrodes and SUA in monkey N. The locations of the recorded SUAs are indicated in the inset grid. The filled triangles in **(C,D)** indicate the space constants at which the amplitude dropped below 37% of its maximum value. The *r*-values of regression in **(C,D)** are 0.85 (*p* = 3.1*e* - 25), 0.88 (*p* = 4.4*e* - 23), and 0.69 (*p* = 2.1*e* - 09), respectively. **(E,F,G)** Map of peak latencies and relationships between distance and latency. Same conventions as in **(B,C,D)**. The *r*-values of regression in **(F,G)** are 0.46 (*p* = 4.7*e* - 6), 0.71 (*p* = 6.4*e* - 12), and 0.27 (*p* = 0.02), respectively.

A stable SUA signal recorded at one electrode allowed us to investigate its response to stimulation applied at each of the other electrodes of the array. This approach is particularly suitable to precisely analyze how the amplitude and the latency of SUA responses vary with the distance between the stimulating and recording electrodes. Peri-stimulus time histograms (PSTHs) aligned to the stimulus onset were used to analyze SUA responses to sICMS (**Figure 3A**). The bin size was set to 0.2 ms and a total of 3000 stimuli was included in each PSTH. The mean and SD of the spike count during baseline (20 ms before stimulus onset) were computed and the following criteria were applied to quantify excitatory and inhibitory responses (Kraskov et al., 2011): the post-stimulus response was considered as excitatory when at least three consecutive bins were above the mean + 2 ^∗^ SD and as inhibitory when at least five consecutive bins were below mean - SD. The response probability – i.e., the total number of spike counts above baseline during the peak period divided by the total number of stimuli – was used to estimate the strength of excitatory connections between stimulating and recording sites. The timing of the peak in the PSTH was used to measure the response latency. The velocity of activity propagation toward the recording site was estimated from the slope of the linear regression of the response latencies with distance.

**FIGURE 4:**
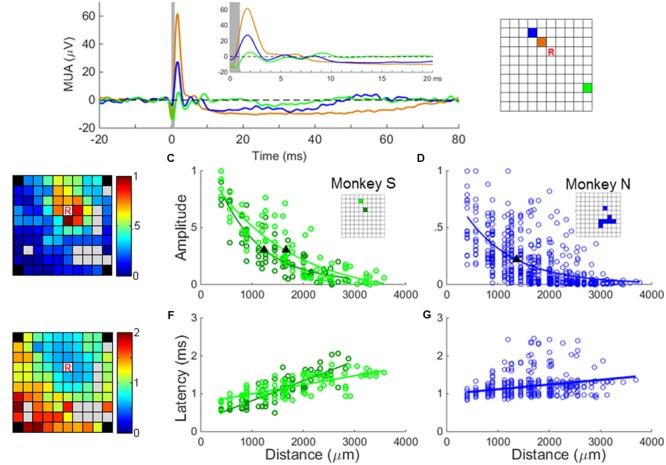
**MUA responses to sICMS. (A)** The averaged MUA responses at one recording site (labeled ‘R’ in the right panel) to stimulation applied successively at three electrodes located at a distance of 0.56 mm (black), 1.12 mm (blue), and 2.24 mm (green), respectively. The responses are aligned to stimulus onset (time 0) and the average baseline activity was subtracted. The artifact blanking period is indicated by the shaded gray area. The inset shows the responses during the first 20 ms. **(B)** The peaks of the normalized MUA responses of channel ‘R’ when stimulating all other channels were mapped on the array dimension (left) and regressed as an exponential decay function of the distances (**C** and **D** for monkey S and N, respectively). The filled triangles in **(C,D)** indicate the space constants of the amplitude decay when the amplitudes dropped to 37% of its origin. The r values of regression in **(C,D)** are 0.88 (*p* = 1.1*e* - 26), 0.86 (*p* = 1.2*e* - 24), and 0.62 (*p* = 3.2*e* - 67), respectively. **(E)** The onset latencies of MUA (when post-stimulus MUA crosses the baseline +3 SD) are mapped and regressed as a linear function of distances (**F** and **G** for monkey S and N, respectively). The *r*-values of regression in **(F,G)** are 0.85 (*p* = 7.2*e* - 25), 0.72 (*p* = 6.1*e* - 15) and 0.39 (*p* = 8.3*e* – 24), respectively.

As it was not possible to isolate on each electrode a stable SUA, we computed a MUA signal at nearly all electrodes of the array. A small subset of channels on which the recorded signal was contaminated by noise was excluded from our analysis. We computed MUA responses by averaging across stimulation sweeps the MUA signals aligned on sICMS onset. Preliminary analysis showed that the MUA responses were similar in latency and amplitude when computed from 500 or 3000 sweeps. Therefore for computation efficiency, only the first 500 sweeps of recordings were used in the analysis. The most common response was characterized by an early peak of excitation followed by a period of inhibition (**Figure 4A**). The MUA response was considered significant when its peak amplitude, measured sweep by sweep, was significantly different from the amplitude measured at a single point 5ms before stimulation onset, during the baseline period (*t*-test, *p* < 0.01). All data used for *t*-test were verified for normality using the Kolmogorov–Smirnov test.

In a first analysis, we investigated if the SUA and MUA responses to sICMS shared similar spatio-temporal properties. To do so, the approach used for the SUA analysis was reproduced with MUA. We measured the amplitude of the MUA response at one electrode while stimulating all other electrodes one by one. We regressed the relationship between amplitude and distance as an exponential function and quantified the spatial spread of the stimulation using the space constant, i.e., the distance at which the response amplitude dropped below 37% of its maximum (**Figures 4B,C**). The relationship between the peak latency of the MUA response and the distance between stimulating and recording electrodes was analyzed in a similar way (**Figures 4D,E**).

**FIGURE 5:**
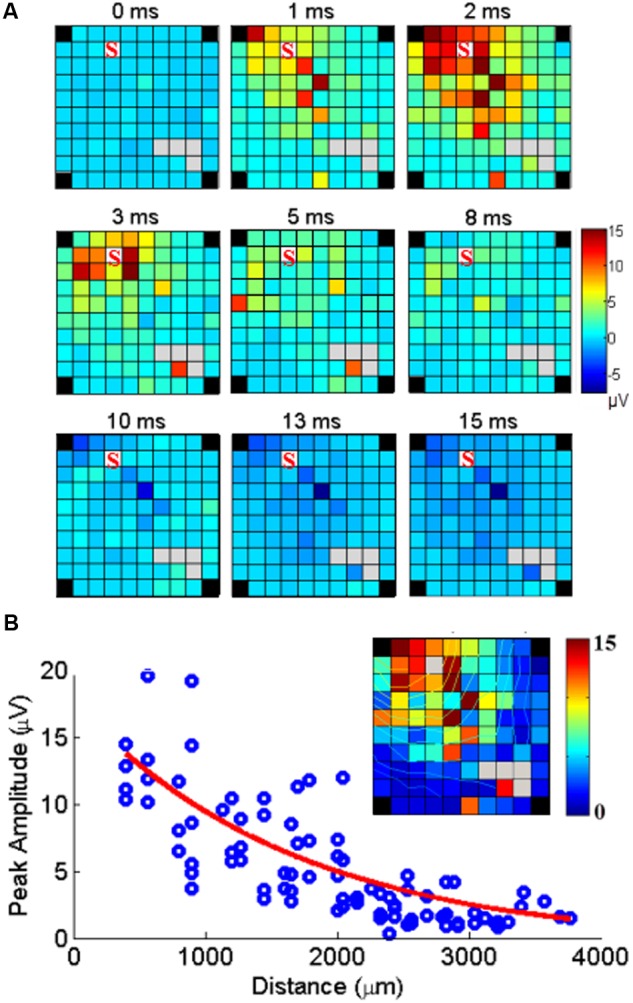
**Cortical spread of MUA responses evoked by stimulation at one electrode. (A)** Example of maps of the MUA peak amplitudes following a single pulse of ICMS at one electrode (marked S) at time 0. The color indicates the MUA peak amplitude at each time point after stimulus onset (0 ms). **(B)** The peak amplitudes of evoked MUA response at each recording channel are regressed as a function of distance. The inset shows the peak responses mapped on the array including isocontour lines.

We then used the MUA signals to explore the spatio-temporal properties of the spread of activity induced by stimulation at one electrode. The spread of activation was mapped onto the 10 × 10 grid of the Utah array, where the amplitude of the response at each electrode is plotted in 1ms time-intervals (**Figure 5A**). The extent of the activity spread was quantified by detecting the electrodes showing a significant MUA response. The inhibitory effects were analyzed in a similar way and the amplitude of excitatory and inhibitory responses at each electrode were compared.

Finally, we investigated if the spread of activity had similar spatial properties when the stimulation was applied at different locations on the array. For this purpose, the map of MUA evoked-effects from each stimulation site was fit with a 2D elliptical Gaussian surface with the free parameters baseline *B*, peak response *A*, major sigma σ_M_, minor sigma σ_m_ and rotation angle 𝜃.

$$f(x,y)=B+A*\exp(-(a{\left(x-x_{0}\right)}^{2}+2b(x-x_{0})(y-y_{0})+c{\left(y-y_{0}\right)}^{2}))$$

in which

$$\begin{array}{c} a=\frac{\cos^{2}\theta }{2\sigma_{M}^{2}}+\frac{\sin^{2}\theta }{2\sigma_{m}^{2}}\\ b=\frac{\sin2\theta }{4\sigma_{M}^{2}}+\frac{\sin2\theta }{4\sigma_{m}^{2}}\\ c=\frac{\sin^{2}\theta }{2\sigma_{M}^{2}}+\frac{\cos^{2}\theta }{2\sigma_{m}^{2}} \end{array}$$

and (*x*_0_, *y*_0_) is the coordinate of the peak response, which was set to the location of the stimulating electrode. The fit ratio (ratio between major and minor sigma σ_M_/σ_m_) was calculated to quantify the direction of the activation spread. If the fit ratio is close to 1, it means that the propagation of the stimulation activity was isotropic.

## Results

We combined sICMS and multielectrode recordings with chronically implanted Utah arrays to investigate the nature of horizontal interactions between distant sites in the motor cortex. We first compared how the SUA and MUA signals recorded at one electrode were modulated with the distance to the stimulating electrode. We then used MUA responses to analyze the spatio-temporal properties of the activity spread following sICMS.

### SUA Responses Evoked by Single Pulse ICMS

**Figure 3A** shows typical SUA responses recorded at one electrode to sICMS applied at 2 distant electrodes. The PSTHs are characterized by a brief excitatory peak occurring at a very short latency (1–2 ms) followed by a longer period of activity suppression. The suppression period was often followed by a rebound of excitation before returning to the baseline level. In some cases, multiple excitatory peaks were observed after the initial peak (right panel in **Figure 3A**). These peaks were usually smaller in amplitude and separated by silent periods of around 3 ms.

In the two monkeys, we first looked for electrodes with stable SUA over several days of recording. The stability of the units was assessed by visual inspection of the spike waveform and ISI histograms. In monkey S, two channels had a very stable SUA with a highly distinctive spike shape across recording sessions. These units were characterized by a large signal to noise ratio of 10.02 and 11.83, respectively, computed with the formula described in Hatsopoulos et al. (2004). This allowed us analyzing their responses to sICMS applied at all other electrodes of the array. We quantified the amplitude of sICMS effects by dividing the number of spikes in the first peak of the PSTH by the total number of sICMS pulses. The duration of this first peak suggests that it included no more than one spike per sweep. Thus, a value of 1 indicates that every single pulse triggered a spike. The map in **Figure 3B** illustrates the amplitude of sICMS effects evoked in one SUA in response to sICMS applied at all other electrodes. The amplitude of the response at the recording site is color-coded at the location of the stimulating electrode. The strongest effects are indicated in red: they were commonly evoked by stimulation of the closest electrodes to the recording electrode, 400 and 565 μm apart, respectively. The evoked effects decreased in amplitude with increasing distance between the stimulating and recording electrode. The plot in **Figure 3C** illustrates the amplitude of sICMS effects in relation to distance for the two stable SUAs of monkey S. For each single unit, the relationship between distance and amplitude is estimated by an exponential function. The measured space constants were 954.3 and 1328.8 μm for these two SUAs.

In monkey N, only 12 electrodes were used as stimulating electrodes. In this monkey, the recording of SUAs was less stable than in monkey S and no SUAs were maintained throughout the stimulation of all electrodes. Therefore for this monkey, the properties of SUA-evoked responses were estimated from all possible SUAs isolated while stimulating one of the 12 stimulation sites (see asterisks in **Figure 1B**). As for monkey S, the relationship between distance and amplitude was estimated by an exponential function (**Figure 3D**). The space constant was 1069 μm for this combination of SUA-evoked responses, comparable to the space constants of monkey S.

The map in **Figure 3E** illustrates the latency of sICMS effects evoked in one stable SUA from monkey S in response to sICMS applied at all other electrodes. Here again, the response-latency is color-coded and is plotted at the location of the stimulating electrode. sICMS effects were evoked at very short latency when stimulation was applied close to the recording electrode and the latency increased with distance. In **Figure 3F**, the latencies of the sICMS effects are plotted as a function of the distance for the two stable SUAs of monkey S. In contrast to the amplitude effects (**Figure 3C**), the latency was linearly related to distance for both SUAs. On average, the latency was around 1 ms at the closest electrode (400 μm) and more than 2 ms at distances above 3000 μm. The velocity estimated from the regression slope in **Figure 3F** was about 3 m/s. Similar results were observed for the combined SUAs of monkey N (**Figure 3G**).

### MUA Responses Evoked by Single Pulse ICMS

The experiments were performed 5 to 6 months after array implantation and stable SUA signals could be recorded only from a small subset of electrodes. In order to generalize the observations made on SUAs, we computed MUAs at each channel following the procedure described in the “Materials and Methods” Section. **Figure 4A** shows the averaged MUA responses recorded at the location R while stimulation was applied at increasing distances from this recording electrode. As for the SUAs, the MUA responses show one or multiple excitatory peaks of short latency, followed by a long period of activity suppression below the baseline level. The excitation lasts around 10 ms, whereas the inhibition can last up to 80 ms at stimulation intensity of 40 μA. The map in **Figure 4B** illustrates the amplitude of the peak MUA-evoked responses measured at the recording electrode while stimulating all other electrodes one by one. The peak amplitude was normalized to the largest response measured across the array after subtraction of the baseline activity. It is color-coded at the location of the stimulating electrode. For each electrode except one (83/84), the average amplitude of the peak in the post-stimulus response was significantly larger than the average baseline activity (*t*-test, *p* < 0.01). The relationship between the peak amplitude and the distance to the stimulating electrode was fitted by an exponential decaying function at two distinct recording locations (**Figure 4C**) leading to a space constant of 1245 μm and 1654 mm, respectively. **Figure 4D** indicates that for monkey N, the relationship between amplitude and distance for all stimulating/recording pairs across all stimulation sessions (*N* = 84 MUAs) shows a similar trend. The velocity (**Figures 4E–G**) estimated from the relationship between distance and latency was 8.03 m/s and 7.29 m/s for monkey S and N, respectively, comparable to the fastest velocities measures at the single unit level.

### Spatial-Temporal Patterns of Stimulus Evoked MU Responses

Since SUA and MUA responses to single pulse ICMS showed very similar properties in terms of space/amplitude and space/latency relationships, we used MUA responses to analyze how the neuronal response triggered by stimulation at one location spreads along the cortical surface. The set of maps in **Figure 5A** illustrates the spatial distribution of MUA responses across the array, at different time points from stimulus onset (*t* = 0 ms) following single pulse stimulations applied at one electrode. The excitatory response reached a maximum amplitude after 2 ms spreading across the entire array. After 8 ms, excitation turned to suppression. **Figure 5B** shows the peak amplitude measured at each recording electrode with respect to the distance between the stimulating and recording electrode. Significant responses were visible up to 4 mm away from the stimulation site. Outlier responses are clearly visible at some electrodes (i.e., the orange channel in the bottom left corner of the array at 3 and 5 ms). Complementary analysis revealed that these responses originate from antidromic SUA responses evoked in every sweep at fixed latency and boosting the MUA measure.

### sICMS-Evoked Excitations and Inhibitions

**FIGURE 6:**
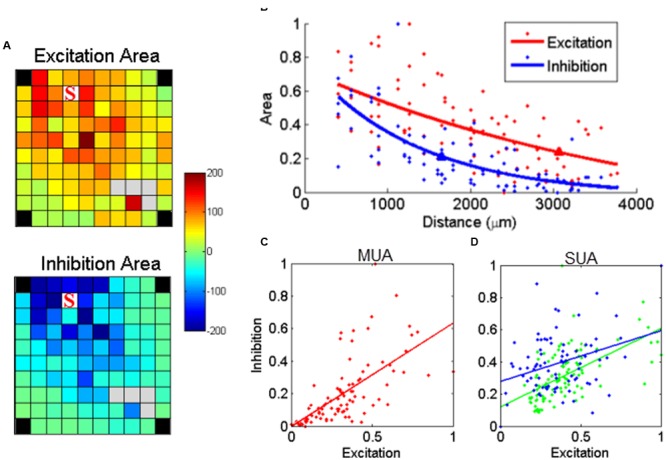
**Relationship between stimulus-evoked excitation and inhibition. (A)** The post-stimulus MUA areas (a.u.) above (excitation) and below (inhibition) the baseline were calculated for each channel and mapped on the array dimension, respectively. The stimulating electrode is labeled with ‘S’. **(B)** The normalized area values for excitation and inhibition were further regressed as a function of distance, respectively. The triangle labeled on each regression line indicated the space constant when the amplitudes dropped to 37% of its origin. **(C)** Stimulus-evoked excitation plotted against the inhibition for MUA. Each dot represents one recording channel in **(A)**. The stimulus evoked inhibition was partially linearly correlated with excitation (*p* = 8.1*e* - 06, *r* = 0.45). **(D)** The linear relationship was also true for all SUAs in both monkeys (*p* = 2*e* - 10 and 1.7*e* - 04 for monkey N and S, respectively).

Besides excitation, sICMS also evoked long periods of activity suppression in both SUA and MUA responses. This suppression is likely to reflect inhibition of neuronal activity at the recording electrodes. To evaluate the relationship between the amplitude of excitation and inhibition, we calculated the area above and below the baseline, i.e., the 20 ms before stimulus onset, in the MUA responses. **Figure 6A** shows the maps for excitation and inhibition for one example electrode. These two maps reveal that both excitation and inhibition spread over long distances from the stimulating electrode, but with a larger extent for excitatory responses. For a more direct comparison of the distance-effect on excitation and inhibition, we normalized the excitation and inhibition areas measured at each electrode by the largest excitation and inhibition areas of the entire array. Both excitation and inhibition decreased with distance, but excitation had a larger space constant than inhibition (**Figure 6B**). We repeated this analysis for all stimulation sites on the array and observed that, on average, excitation had a significant larger space constant than inhibition (2034 ± 778 vs. 1674 ± 417 μm, *t*-test, *p* < 0.01). Despite this difference, **Figure 6C** demonstrates that excitation and inhibition were linearly related. This linear relationship might be due to the fact that both excitation and inhibition are individually correlated with distance. Therefore, in order to analyze the relationship between excitation and inhibition independently from distance, we used a partial correlation measure by removing the factor distance.

The linear relationship between excitation and inhibition remained significant (*p* < 0.01), indicating that the strength of inhibition depends on the intensity of the preceding excitation. We used the same approach to analyze the relationship between excitation and inhibition for the two stable SUAs in monkey S. As shown in **Figure 6D**, the linear regression in both SUAs demonstrates the balance between excitation and inhibition at the single unit level.

### Effect of Stimulation Intensity on sICMS Evoked Responses

**FIGURE 7:**
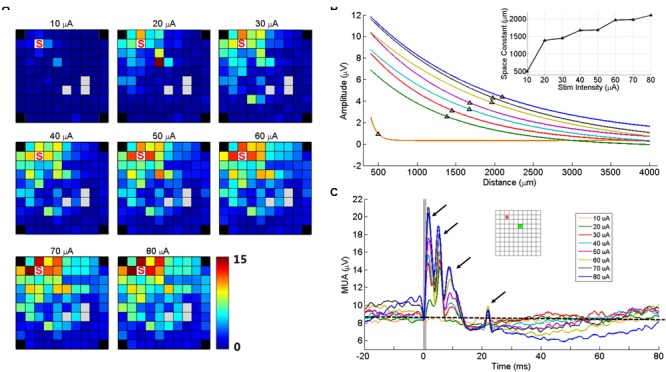
**Stimulation intensity effects on MUA responses. (A)** Maps of peak amplitudes in response to sICMS at electrode ‘S’ at different stimulation intensities (10–80 μA). **(B)** Regression lines of the MUA responses with distance for different stimulus intensities. The black triangles on each line indicate the space constant when the amplitudes dropped to 37% of its origin. The inset shows the space constant as a function of stimulus intensity. **(C)** The MUA traces recorded at one example electrode (green square in the inset) for different intensities, aligned to stimulus onset. The arrows show the four peaks of the responses evoked at high stimulus intensity. The horizontal dashed line indicates the baseline activity level at 10 μA. Note that at high intensities, the prolonged inhibition is followed by a rebound in excitation altering the baseline at stimulation onset.

Two electrodes were used to test the effect of changes in stimulation intensity on sICMS evoked responses. As shown in **Figure 7A** for one of these two electrodes, the peak amplitude of the response at each recording electrode increased when stimulation intensity increased from 10 to 80 μA. In **Figure 7B**, the peak response at each electrode was regressed as a function of distance for different intensities and the space constants were computed. The space constant increased strongly between 10 and 20 μA and more gradually up to 80 μA. For stimulation intensities >20 μA, the space constant increased by about 12 μm/μA.

**Figure 7C** shows the MUA responses recorded at one electrode (green square) to stimulation of increasing intensities at another electrode (red square). At this (green) electrode, the stimulation evoked four response peaks at high intensity. However the last two peaks were absent for intensities below 30 μA. Visual inspection of SUA responses on this electrode revealed that the fourth peak was actually caused by the antidromic or orthodromic activation as the responses were at a very fixed latency. We also observed that the duration of inhibitory responses increased with intensity, indicating the balance between excitation and inhibition. In the stimulations of high intensities, the MUA did not return to baseline with such the 100 ms inter-stimulus interval.

### Cortical Spread of sICMS-Effects from Different Cortical Locations

**FIGURE 8:**
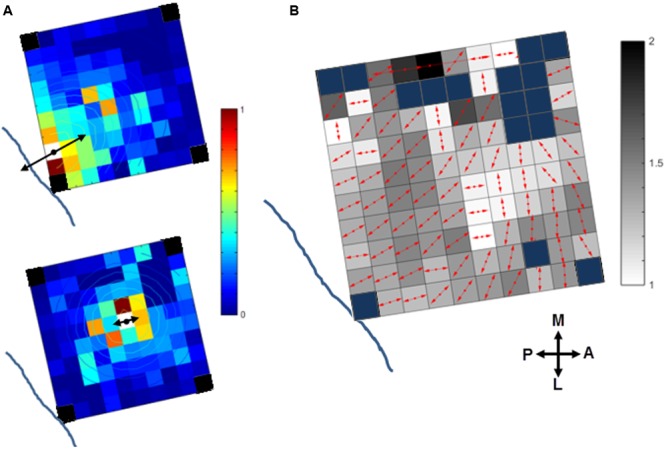
**Orientation of the stimulus-evoked propagation of MUA in monkey S. (A)** The contour plot of the 2D Gaussian fit is overlapping with the MUA activity spread when stimulating two distinct channels (white squares) close to the central sulcus (top) or at the array center (bottom). The dominant orientation of the spread (fit angle) and its strength (fit ratio) are indicated at the stimulation site by the double-sided black arrows. The blue lines indicates the location of the central sulcus (see **Figure 1A**, right panel for monkey S). Note, the four corner electrodes were not connected (black squares). **(B)** Summary map of activity spread for all the stimulated electrodes. The red arrows at each electrode indicate the fit angle and the underlying gray level scales the fit ratio. Blue squares: rejected channels due to noise contamination in the recorded signals or not connected.

We investigated if the cortical spread of activity induced by sICMS was stronger and had a larger extent along preferred orientations in motor cortex. This analysis was performed in monkey S only. The maps of MUA responses to stimulation applied at each electrode were fit with a 2D elliptical Gaussian to find out if MUA propagates along a preferred orientation and to evaluate the strength of the orientation bias. The Gaussian fit obtained from the MUA responses to sICMS at two distinct electrodes is shown in **Figure 8A**. The contour lines show the results of the fitting. The arrow originating from the stimulated electrode represents the main axis of the Gaussian fit (fitting angle) and indicates the main orientation of the activity spread. The length of the arrow indicates the ratio between the long and short axis (perpendicular to each other) of the Gaussian fits and signals the strength of the orientation bias. A ratio of 1 represents an isotropic spread of similar strength in all orientations. The stimulation applied close to the central sulcus (**Figure 8A**, top) evoked a spread with clear orientation bias (fitting ratio 1.48) perpendicular to the central sulcus. Alternatively, the stimulation at the center of the array (**Figure 8A**, bottom) evoked an almost isotropic spread of activity (fitting ratio, 1.03). The fitting results for all electrodes are presented in **Figure 8B** on an overlapped map in which the arrow at each electrode indicates the fitting angle and the underlying gray scale (16 levels) the fitting ratio. For **Figure 8B**, the data were smoothed over the array by averaging the map obtained from each stimulating electrode with those obtained from all directly adjacent electrodes, after spatial alignment of the stimulating electrodes. The fitting angle and fitting ratio were computed from the smoothed map at each electrode, one by one. Light and dark squares represent isotropic and anistropic spreads, respectively. For the posterior electrodes, the dominant orientation of the spread was orthogonal to the central sulcus, along a rostral-caudal axis. The orientation bias was less pronounced at the most anterior electrodes and was mainly aligned along the medio-lateral axis, parallel to the central sulcus.

These preferential orientations can be related to the underlying somatotopic organization of the motor cortex, as illustrated in **Figure 1B** (right panel for monkey S). The comparison of these figures suggests that sICMS effects spread could be partly driven by the underlying connectivity between arm and hand related body representations.

### Spread of Stimulus-Evoked Activity from Different Body Representations

**FIGURE 9:**
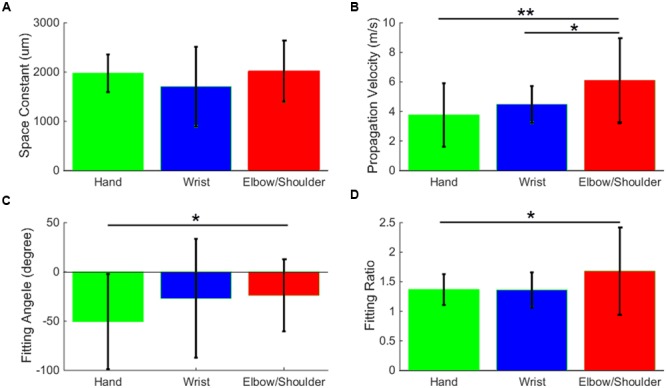
**Stimulation-evoked spread of MUA activity from distinct body representations.** The space constant **(A)**, propagation velocity **(B)**, fitting angle **(C)**, and fitting ratio **(D)** are averaged and bar-plotted for the three groups of channels, hand (green), wrist (blue), and elbow/shoulder (red). All values are compared with *t*-tests and significance levels ^∗^ (*p* < 0.05) and ^∗∗^ (*p* < 0.01) are plotted.

In the last part of the analysis, we used the data from monkey S to compare the spread of activity evoked by sICMS applied in the different body representations of the motor cortex. The results of this analysis are summarized in **Figure 9**. The electrodes evoking multi-joint responses in the ICMS mapping procedure were excluded from this part of the analysis. The space constants did not differ between the three groups of channels (hand, wrist, and elbow/shoulder) suggesting that the spatial extent of horizontal connectivity is similar in the different somatotopic representations. However, the propagation velocities (**Figure 9B**) determined from elbow/shoulder channels were significantly higher than those from hand (*p* < 0.01) and wrist channels (*p* < 0.05), suggesting a more dense and direct connectivity within the territory covered by the array starting from elbow/shoulder locations than from hand locations. We additionally used the data presented in **Figure 8B** to compare the average fitting angle and fitting ratio in the three body representations. The fitting ratio (**Figure 9D**) was larger from elbow/shoulder related channels than from wrist or digit related channels, indicating stronger anisotropies of activity spread in the proximal representation. Finally, the fitting angle (**Figure 9C**) was significantly different between hand and elbow/shoulder-related sites. The average fitting angle in the elbow/shoulder channels confirms that, in this body representation, the spread tend to align along the medio-lateral axis. On the other hand, the average fitting angle in the hand channels indicates a spread of activity along the rostral-caudal axis.

## Discussion

We combined sICMS and intracortical recordings with chronically implanted multielectrode arrays to explore the pattern of functional connectivity in monkey motor cortex. We first provided the proof of concept that multi-electrode arrays can be used to apply intracortical micro-stimulation at one electrode while recording SUAs and MUAs, respectively, at other electrodes within 1 ms of stimulation onset. With this approach, we observed that SUA and MUA responses to sICMS present very similar properties. They are both characterized by a peak of excitation followed by a period of activity suppression. They also show similar modulations in amplitude and latency as a function of the distance between the stimulating and recording electrodes. We therefore used MUA responses to thoroughly characterize the spatio-temporal properties of activity propagation following sICMS applied at different locations on the array. Our analysis provided three main results. First, we observed that inhibitory responses spread at shorter distances than excitatory ones. Second, we showed that increasing the intensity of stimulation moderately increases the area of the cortical territory recruited by the stimulation. Third, the propagation of activity showed some local anisotropies, being preferentially aligned along the rostro-caudal orientation close to the central sulcus. Altogether, these findings provide new insights into the intrinsic organization of neuronal networks in the motor cortex.

### Stimulation/Recording Switch Enables Recording Soon after Stimulation

Microstimulation has long been used for functional mapping of cortical area and its direct and indirect evoked response has been observed using various techniques, including fMRI (Tolias et al., 2005; Logothetis et al., 2010; Matsui et al., 2012), optical imaging (Seidemann et al., 2002; Brock et al., 2013), calcium signal (Histed et al., 2009), and electrophysiology (Stoney et al., 1968; Matsumura et al., 1996; Baker et al., 1998; Butovas and Schwarz, 2003). The main disadvantage of the electrophysiological approach is the presence of a large stimulation artifact that interferes with the neuronal signal right after stimulation. The artifact is partly due to the capacitive coupling between the stimulating and recording electrodes (McGill et al., 1982). At the end of a stimulation pulse, the accumulated charges in the stimulating electrode are dissipated through the electrode/tissue interface (Merrill et al., 2005) which causes a long exponentially decaying potential at the recording electrodes. As the interface has a large capacitive component, the time constant of the decay can exceed several milliseconds (Jimbo et al., 2003; Merrill et al., 2005) and prevent the measure of evoked-responses occurring at short latency (**Figure 2A**, red trace). In this study, we were able to record neuronal activity within 1 ms after stimulus onset (**Figure 2B**) by forcing the recorded signal rapidly back to baseline after stimulation (**Figure 2A**). This was achieved by switching all electrodes from recording mode to stimulation mode before stimulus onset and switching them back to recording mode right after stimulus offset. The switching helped in two ways: (1) At the recording electrodes, it protected the amplifier from being exposed to high stimulation current during stimulation and therefore limits long lasting saturation effects and (2) at the stimulating electrode, it accelerated the discharging process by switching from the high impedance stimulating circuits to the lower impedance recording circuits. This operation allowed for extremely fast discharging of the stimulating electrode, and brought the signal back to baseline to enable neuronal recordings soon after stimulation.

### SUA and MUA Responses to sICMS Share Similar Properties

Following electrode switch, we were able to detect clean SUA signals within less than 0.5 ms after stimulus offset. In agreement with previous studies (Butovas and Schwarz, 2003; Kraskov et al., 2011; Kunori et al., 2014), we showed that SUA responses to sICMS are characterized by a short peak of excitation followed by a long period of inhibition. We assume that the excitatory response reflects the indirect *trans*-synaptic activation of the recorded neurons by the stimulation pulse. First, the excitation peak has a typical duration of 1–2 ms reflecting some jittering in the response latency. Second, the SUA response is probabilistic in nature and only triggered by a fraction of the stimulation pulses. Therefore, such response is likely to be driven by synaptic projections originating from the neurons and axons located close to the stimulating electrode and directly activated by the stimulation pulse. Our data show that sICMS can evoke single unit responses with high probability even at electrodes located several millimeters away from the stimulation site. This result contrasts with the very low probability of finding putative synaptic connections between single units (0.17%) and the fact that these connections are most often (76%) observed between neurons recorded at the same electrode (Peyrache et al., 2012). The powerful effect of sICMS in driving single unit responses is assumed to be due to the synchronized activation of many neurons and axons en-passant around the stimulated electrode that synaptically activate neurons located at distant sites.

We showed that for a given SUA, the amplitude of the excitatory response exponentially decreases and its latency linearly increases with increasing distance to the stimulating electrode. This observation closely matches the observation of activity propagation evoked by single spikes in the visual cortex of monkeys whose amplitude exponentially decays in relation to distance with a space constant of around 2 mm (Nauhaus et al., 2009). It is also in line with the fact that in the motor cortex, the density of synaptic boutons along horizontal axon collaterals that originate from a given cortical location is highest within a 1.5 mm radius and decreases monotonically with distance (Capaday et al., 2009). The estimated velocity from the distance/latency regression (3 m/s) suggests that the activity propagates along axons of average size. Interestingly, this velocity of propagation is an order of magnitude larger than the propagation velocity of traveling waves in the cortex of awake monkeys (Rubino et al., 2006; Nauhaus et al., 2009; Muller et al., 2014) or the velocity of activity spread triggered by local injection of bicuculline methochloride, a GABAa antagonist (Capaday et al., 2011). This discrepancy suggests that the cortical network is differently recruited by the highly synchronized activation of neurons that follows sICMS than by the physiological activation of neurons through synaptic excitation.

As for SUA, MUA responses to sICMS show a typical pattern of response characterized by a brief peak of excitation followed by a longer period of inhibition. These responses are strongly modulated by the distance between the stimulation and recording electrodes. The average space constant measured from MUA and SUA responses are in a similar range but the velocity of propagation is noticeably faster when estimated from MUA (around 10 m/s). This latter observation fits with the fact that the latency of MUA response is driven by the propagation velocity of the largest axons. Altogether, these results suggest that MUA and SUA responses are closely related and that MUA is appropriate to analyze the spatial properties of activity spread in the motor cortex.

### Differential Effect of sICMS on Excitatory and Inhibitory MUA Responses

We therefore used MUA recordings to characterize the pattern of neural propagation evoked by the sICMS applied at a single electrode. **Figure 5A** shows that following the stimulation pulse, the excitatory response propagates rapidly to a large range of electrodes, reaches a maximum extent within 2–3 ms before gradually turning to a longer period of inhibition. A quantitative analysis of the relationship between excitation and inhibition showed that they are both decreasing with the distance and correlating with each other, but the spatial spread of excitation is significantly larger than of inhibition (**Figure 6**). This difference is likely to reflect different properties of the underlying neuronal network. It has been demonstrated that in rodents, most inhibitory neurons have short axonal projections to neurons within a volume of 0.4 mm radius (Fino and Yuste, 2011; Isaacson and Scanziani, 2011), and are not expected to carry inhibitory signals to distant sites. In agreement with these observations, we assume that inhibitory responses are mediated by excitatory neurons carrying information from the stimulation site to the recording site where they excite local inhibitory networks. Due to additional synaptic delays, this indirect inhibition occurs after the excitatory response and is more attenuated at distant sites. Also, the recurrence of inhibitory networks (Kapfer et al., 2007) produces a long-lasting inhibition in MUA recordings. Previous studies have shown that such polysynaptic inhibition is reduced by pharmacological blockade of GABA-A (Silberberg and Markram, 2007; Zhu et al., 2011) or GABA-B receptors (Butovas et al., 2006) and weakened and more imprecise in connexin 36 knockout (KO) mice in which gap junctions of inhibitory networks are not functional (Butovas et al., 2006) suggesting complementary roles of these transmission mechanisms in shaping the inhibitory response.

### Changes in sICMS Intensity Has Moderate Effects on Activity Spread

We observed that the stimulation intensity influences the spread of stimulus-evoked excitation. Previous estimates suggest that with an average excitability constant of 1,292 μA/mm^2^, increasing the stimulation intensity from 20 to 80 μA increases the radius of directly activated neurons from 124 to 249 μm (Stoney et al., 1968; Tehovnik, 1996; Tehovnik et al., 2006), corresponding to a tenfold increase of the stimulated volume. These changes result in a marked increase of the response amplitude at all responding electrodes reflected in the larger space constants (**Figure 7B**). However, the maps in **Figure 7A** indicate that a large number of distant electrodes that were not responsive at 20 μA were also not responsive to 80 μA. These observations suggest that although a larger population of neurons is directly stimulated at higher intensity of stimulation, the maximal distance of the cortical spread remains limited by the maximum reach distance of axon collaterals around 3–4 mm.

Increasing the stimulation intensity also changes the pattern of MUA response at the recording electrodes. For stimulation intensities >20 μA, we observed multiple peaks in the MUA recordings. These multiple peaks are likely to originate from recurrent activation of SUA as shown in **Figure 3A**. Multiple peaks in MI SUA have been observed by Kraskov et al. (2011) in response to stimulation of the ventral premotor cortex suggesting that long distance interactions between distinct motor areas are involving very similar pathways.

### The Spread of sICMS Effects Reveals the Spatial Properties of Intracortical Connectivity

The Gaussian fitting of MUA responses revealed that the spatial properties of neural spread varied with the location of the stimulating electrode on the array. More specifically, the neural spread was anisoptropic when sICMS was applied close to the central sulcus (hand-related channels) with an orientation bias aligned along the rostral-caudal axis. The spread was also anisotropic following stimulation of the most anterior electrodes (elbow/shoulder-channels) but with an orientation bias along the medio-lateral axis. We have shown that this difference in orientation is statistically significant between digit and elbow/shoulder-related channels. In addition, our analysis showed that the spread from elbow/shoulder-related channels has a similar space constant but a significantly larger velocity than the spread from hand or wrist channels. This result suggests that the horizontal connections originating from elbow/shoulder-related sites are more dense and direct but have a similar extent than the connections from other body representations of the motor cortex. Finally, stimulation of the center electrodes evoked an isotropic spread. This pattern of propagation shows some striking similarities with the pattern of propagation in LFP beta waves reported by Rubino et al. (2006). These waves are predominantly aligned along the rostro-caudal axis close the central sulcus and along the medio-lateral axis in the dorsal premotor cortex (their **Figure 5**). Our data support the hypothesis that the dominant propagation axes of sICMS evoked effects relate to the underlying horizontal connectivity in the motor cortex.

It could be argued that some of the observed effects were due to some instability in the recordings and some uncontrolled displacement of the array on the cortical surface. However, we assume that this is unlikely for two main reasons. First, the maps of ICMS-evoked body movements performed at 3 months intervals are strikingly similar (**Figure 1B**). Second, the sICMS data presented in this study were collected over a 10- and 6-day period for monkey S and N, respectively, periods during which a massive reorganization of the cortex is unlikely to occur.

Previous anatomical studies have shown that horizontal axon collaterals preferentially linked representation zones of the same body part in motor cortex (Huntley and Jones, 1991; Keller, 1993). Within the upper limb representation, labeled cell bodies tend to be aligned along the rostro-caudal axis following HRP injection close to the central sulcus (Huntley and Jones, 1991). The preferred rostro-caudal orientation of horizontal connections has been also reported using histological (Gatter et al., 1978) and electrophysiological (Godschalk et al., 1984) approaches. In a similar way, the study of Capaday et al. (2009) suggests that in cat motor cortex, the distribution of synaptic boutons along axon collaterals follows preferential orientation around the cruciate sulcus, even though this spatial bias was not quantified systematically. In keeping with all these observations, our approach provides additional evidence that the spatio-temporal dynamics of motor cortex activation is partly determined by the underlying neural connectivity. It also fits with the idea that the motor cortex is not functionally homogeneous but forms a complex network of interacting subregions (Dea et al., 2016).

## Ethics Statement

This study was approved by the local ethical committee in Neuroscience, C2EA – 71, Marseille, France.

## Author Contributions

YH and TB conceived and designed the research; YH and TB performed the experiments; YH and TB analyzed the data; YH, AR, and TB interpreted results of experiment; YH prepared figures; YH and TB drafted the manuscript; YH, AR, and TB edited and revised the manuscript; YH, AR, and TB approved the final version of manuscript.

## Conflict of Interest Statement

The authors declare that the research was conducted in the absence of any commercial or financial relationships that could be construed as a potential conflict of interest.
